# De Novo Genome Assembly and Comparative Genome Analysis of the Novel Human Fungal Pathogen *Trichosporon austroamericanum* Type-Strain CBS 17435

**DOI:** 10.1007/s11046-025-00942-w

**Published:** 2025-04-04

**Authors:** Elaine C. Francisco, Marie Desnos-Ollivier, Bert Gerrits van den Ende, Ferry Hagen

**Affiliations:** 1 Department of Medical Mycology, Westerdijk Fungal Biodiversity Institute (WI-KNAW), Uppsalalaan 8, 3584CT Utrecht, The Netherlands; 2 Division of Infectious Diseases, Escola Paulista de Medicina-Universidade Federal de São Paulo, São Paulo, Brazil; 3 Center for Monitoring Antimicrobial Resistance Patterns and Genetic Diversity in Clinical and Environmental Isolates from Brazilian Biomes, Universidade Federal do Paraná, Curitiba, Brazil; 4 National Reference Center for Invasive Mycoses and Antifungals, Mycology Translational Research Group, Mycology Department, Institut Pasteur, Université de Paris Cité, Paris, France; 5 Institute for Biodiversity and Ecosystem Dynamics (IBED), University of Amsterdam, Amsterdam, The Netherlands; 6 Department of Medical Microbiology, University Medical Center Utrecht, Utrecht, The Netherlands; 7 Antimicrobial Resistance Institute of São Paulo (ARIES), São Paulo, Brazil

**Keywords:** Rare yeasts, Emerging pathogen, Nanopore sequencing, Genome assembly, *Trichosporon austroamericanum*, *Trichosporon inkin*

## Abstract

*Trichosporon austroamericanum* is a recently described species recognized for its emerging clinical significance in invasive trichosporonosis. In this study, we present the nanopore long-read-based de novo genome assembly of the type-strain CBS 17435. Additionally, we performed genomic comparative analyses with its closest relative, *Trichosporon inkin*.

*Trichosporon austroamericanum* is a recently recognized emerging pathogen, noted for its clinical relevance in causing a range of infections, including both superficial and invasive trichosporonosis [1]. The first case of *T. austroamericanum* was identified during an epidemiological survey in 2013, when it was isolated from a urine sample of a Brazilian kidney transplant recipient. However, sequence analyses from retrospective studies and genomic databases have documented the presence of this species in Europe, Asia, and Latin America [1, 2]. Phylogenetic analyses indicated that this species is most closely related to *Trichosporon inkin.* Notably, *T. austroamericanum* exhibits a range of physiological characteristics, including the ability to grow at 45 °C, that differentiates it from other *Trichosporon* species [1].

We aimed to perform long-read nanopore sequencing and genomic analysis of the *T. austroamericanum* type-strain CBS 17435. This strain was subcultured onto 2% glucose, 50% yeast-extract water, 0.5% bacteriological peptone, 2% technical agar #2 (GYPA), supplemented with 0.5 M sodium chloride to reduce the excessive formation of extracellular polysaccharides, which otherwise negatively impact genomic DNA purification. The previously reported detailed protocol for high-quality genomic DNA purification was followed, with the modification of using proteinase K from Roche Diagnostics (Mannheim, Germany) [3]. The quality and quantity of genomic DNA was assessed using the Qubit in combination with the High Sensitivity kit (ThermoFisher, Waltham, MA, U.S.A.), and by 0.8% agarose gel electrophoresis.

One microgram of gDNA was used as starting point for the library preparation using the multiplex native ligation kit (SQK-NBD114.24; ONT, Oxford, United Kingdom) following the manufacturer’s instructions (protocol version NBE_9169_v114_revQ_15Sep2022, last updated February 16, 2024). The library was loaded onto a MinION R10.4.1 flow cell and raw data was collected using the GridION platform (software release 24.02.16; ONT). Basecalling was performed using Dorado (basecall_model_version_id = dna_r10.4.1_e8.2_400bps_hac@v4.3.0; ONT). Reads with a length ≥ 1000 bp and a quality-score (Q) of ≥ 10 were collected into a single FASTQ file for downstream analyses.

FASTQ data was subjected to an additional quality check using chopper v0.7.0 to collect reads with a length of ≥ 1100 bp and a ≥ Q10, followed by removal of an arbitrary 50 bp from the 5’- and 3’-ends [4]. Thereafter Flye v2.9.3-b1797 was used to generate the de novo genome assembly which was subsequently manually curated [5]. The haploid nuclear genome was found to be 20,968,827 bp in size, comprising eight fragments measuring 3,928,915; 3,876,917; 3,401,721; 2,954,482; 2,383,423; 2,186,886; 1,418,410; and 818,073 bp in length. The circular mitochondrial genome was 35,357 bp in length. Coverage of the nuclear genome was 79X, while the mitochondrial genome had a coverage of 3,479X. The haploid genome size of *T. austroamericanum* CBS 17435 closely matches that of *T. inkin* JCM 9195 (= CBS 5585), which has a haploid genome size of 20.35 Mbp long [6]. Both species have a rather decreased genome size compared to other haploid species in the *Trichosporonales*, which have an average genome size of 23.73 Mbp (± 4.38 Mbp; range 17.23–36.62 Mbp) [6]. The mitochondrial genome of *T. inkin* was recently determined to be 39,466 bp in length, and that of the more distantly related *Trichosporonales* species *Apiotrichum gamsii* and *Apiotrichum gracile* were 38,096 and 34,648 bp in length, respectively [7, 8]. The mitochondrial genome length of 35,357 bp reported here for *T. austroamericanum* is within the observed range of *Trichosporonales* species.

To assess the quality of the de novo genome sequence, a BUSCO v5.8.0 analysis was performed, that yield with the eukaryota_odb10 database 96.5% complete (95.7% single, 0.8% duplicated), 2.7% fragmented, and 0.8% missing BUSCO’s among 255 tested, with the tremellomycetes_odb10 database this was 92.7% (92.3%, 0.4%), 1.4%, and 6.0%, respectively, of the 4,284 BUSCO’s tested [9]. As a comparison, similar BUSCO analysis was done for the *T. inkin* reference genome of JCM 9195 (= CBS 5585) that was retrieved from NCBI Genome (accession number GCA_040365635.1, version April 4, 2024). This yielded comparable values, for the eukaryota_odb10 database 96.9% complete (96.1% single, 0.8% duplicated), 2.4% fragmented, and 0.8% missing genes, and for the tremellomycetes_odb10 database 92.4% (92.0%, 0.4%), 1.5%, and 6.1%, respectively. Additionally, compleasm v0.2.6 was run for the tremellomycetes_odb10 database and resulted in higher completeness scores, with 94.31% complete (94.19% single, 0.12% duplicated), 1% fragmented, and 4.69% missing BUSCO’s for the genome of *T. austroamericanum* CBS 17435, and 94% complete (93.84% single, 0.16% duplicated), 1.07% fragmented, and 4.93% missing BUSCO’s for the genome of *T. inkin* JCM 9195 [10].

We used the web-based deep learning tool Helixer v0.3.4 to predict the number of genes for CBS 17435 and JCM 9195, which were found to be 8,275 and 8,395 genes, respectively [11]. The latter represents a 9–24.1% increase compared to the previously reported 6,766–7,700 predicted genes, which were obtained using the tools Augustus and GeneMark-ES based on data from the *Cryptococcus neoformans* reference genome [6, 12].

The GC% of the nuclear genome of *T. austroamericanum* CBS 17435 was calculated to be 61.38%, comparable to the 63% of *T. inkin* JCM 9195. The GC% of the mitochondrial genome of CBS 17435 was found to be 27.11%, nearly similar to the 27.56% of that of JCM 9195 [8]. The average nucleotide identity (ANI) between the genomes of the *T. austroamericanum* and *T. inkin* type strains was calculated by OrthoANI using the USEARCH algorithm [13]. This analysis returned an OrthoANI value of 84.6472% between the genomes of CBS 17435 and JCM 9195. The ANI between this two *Trichosporon* species is comparable to the ~ 82% previously reported for members of the genus *Cutaneotrichosporon*, as well as to the ANI of 84.73% between *Cutaneotrichosporon oleaginosus* and *Apiotrichum akiyoshidainum* [14, 15]. These ANI values fall well below the 95% threshold recently proposed for species delineation in bacteria based on genome data, which correlates with the historical species delineation criterion of 70% similarity in DNA-DNA hybridization. The ANI reported here for the closely related siblings *T. austroamericanum* and *T. inkin* further supports their distinction as separate species.

## Data Availability

Genomic data has been deposited in NCBI repositories under the following accession numbers: BioProject PRJNA1124242, BioSample SAMN41846244, Sequence Read Archive SRR30989370, and Genome JBIEOS000000000.
